# Analysis of Gene Expression and Proteomic Profiles of Clonal Genotypes from *Theobroma cacao* Subjected to Soil Flooding

**DOI:** 10.1371/journal.pone.0108705

**Published:** 2014-10-07

**Authors:** Fabiana Z. Bertolde, Alex-Alan F. Almeida, Carlos P. Pirovani

**Affiliations:** 1 Instituto Federal de Educação Ciência e Tecnologia, Campus Eunápolis, Eunápolis, Bahia, Brazil; 2 Departamento de Ciências Biológicas, Universidade Estadual de Santa Cruz, Ilhéus, Bahia, Brazil; Universidade Federal do Rio Grande do Sul, Brazil

## Abstract

Soil flooding causes changes in gene transcription, synthesis and degradation of proteins and cell metabolism. The main objective of this study was to understand the biological events of *Theobroma cacao* during soil flooding-induced stress, using the analyses of gene expression and activity of key enzymes involved in fermentation, as well as the identification of differentially expressed proteins by mass spectrometry in two contrasting genotypes for flooding tolerance (tolerant - TSA-792 and susceptible - TSH-774). Soil anoxia caused by flooding has led to changes in the expression pattern of genes associated with the biosynthesis of alcohol dehydrogenase (ADH), pyruvate decarboxylase (PDC) and lactate dehydrogenase (LDH) in leaves and roots of the two evaluated genotypes. Significant differences were observed between the enzyme activities of the two genotypes. Leaves and roots of the TSA-792 genotype showed higher ADH activity as compared to the TSH-774 genotype, whereas the activities of PDC and LDH have varied over the 96 h of soil flooding, being higher for TSA-792 genotype, at the initial stage, and TSH-774 genotype, at the final stage. Some of the identified proteins are those typical of the anaerobic metabolism-involved in glycolysis and alcoholic fermentation-and different proteins associated with photosynthesis, protein metabolism and oxidative stress. The ability to maintain glycolysis and induce fermentation was observed to play an important role in anoxia tolerance in cacao and may also serve to distinguish tolerant and susceptible genotypes in relation to this stressor.

## Introduction

Cocoa, *Theobroma cacao* L. (Malvaceae) is a tropical woody species from the South American tropical rainforest [1]. Its cultivation is primarily engaged in providing almonds that are used in the production of chocolate and other derivatives and by-products that can be processed into cosmetics, jellies, ice creams and juices [2], [3]. *T. cacao* production is seriously affected by different forms of biotic stress, such as fungal diseases and insect attacks [4], and abiotic factors such as irradiance, droughts and floods [5].

Soil flooding is common in some cocoa regions of Brazil, Ghana, Nigeria and Ivory Coast, where precipitation exceeds evapotranspiration; associated with land drainage problems, this condition leads to O_2_ scarcity in the soil [5]. These stress conditions induce plants to a decrease in ATP production by aerobic respiration, resulting in lower growth rate and decreased yields [6]. Adaptations to anoxia in flooded soils involve a combination of morphological and metabolic processes that majorly involve enzymatic systems [7], [8].

One of the major effects of soil flooding is the deprivation of O_2_ in the root zone (anoxia), which can be explained by the slow diffusion of gas in water-saturated soils, about 10,000 times slower than in air [9]. O_2_ deficiency causes rapid changes in gene transcription, protein synthesis and degradation, and cellular metabolism [10]. Under these conditions, the aerobic protein synthesis is blocked and there is induction of anaerobic proteins (ANPs) [11]. Besides from enzymes of glycolytic and fermentative pathways, ANPs include numerous proteins, suggesting the activation of different metabolic responses associated with the adaptation of energy metabolism [12].

O_2_ deficiency in roots inhibits aerobic respiration and induces fermentative pathways [13]. Three key enzymes are involved in fermentation: alcohol dehydrogenase (ADH; E.C. 1.1.1.1), pyruvate decarboxylase (PDC; E.C.4.1.1.17) and lactate dehydrogenase (LDH; E.C. 1.1.1.27). PDC catalyzes the decarboxylation of pyruvate so as to produce carbon dioxide and acetaldehyde, whereas ADH catalyzes ethanol-acetaldehyde oxidation-reduction and NAD^+^ regeneration and LDH catalyzes lactate formation and NAD^+^ regeneration [10]. Even in small quantities, these pathways maintain energy generation (ATP) and thereby ensure the survival of plants subjected to temporary floods. Alcoholic fermentation has been described as the main route for NAD^+^ regeneration under anaerobic conditions [14]. The produced ethanol can easily spread throughout plant tissues, whereas in lactic fermentation, there is lactic acid accumulation, which results in cytoplasmic acidosis and toxicity. Thus, the regulation of cytoplasmic pH is essential for the survival of plants growing in waterlogged conditions [15].

Studies of the molecular responses of *T. cacao* to soil flooding are still scarce. Knowledge of the mechanisms of survival to anoxia is restricted, in large measure, to few species, such as *Oryza sativa* [16], [17], *Zea mays* [18], *Arabidopsis* [19], [20], *Glycine max* [21], [22], and has been based on genomic and proteomic approaches. The main objective of this study was to understand the biological events of *T. cacao* during soil flooding-induced stress through the analyses of gene expression and activity of key enzymes involved in fermentation, as well as the identification of differentially expressed proteins in two genotypes previously identified as tolerant (TSA-792) and susceptible (TSH-774) to soil flooding [23], [24]. In *T. cacao* genotype tolerant to flooding observed many changes in metabolic pathways needed for the maintenance of the production of energy in the condition of O_2_ deficiency and subsequent plant survival.

## Results

### Gene expression of enzymes ADH, PDC and LDH to soil flooding

**Figure 1:**
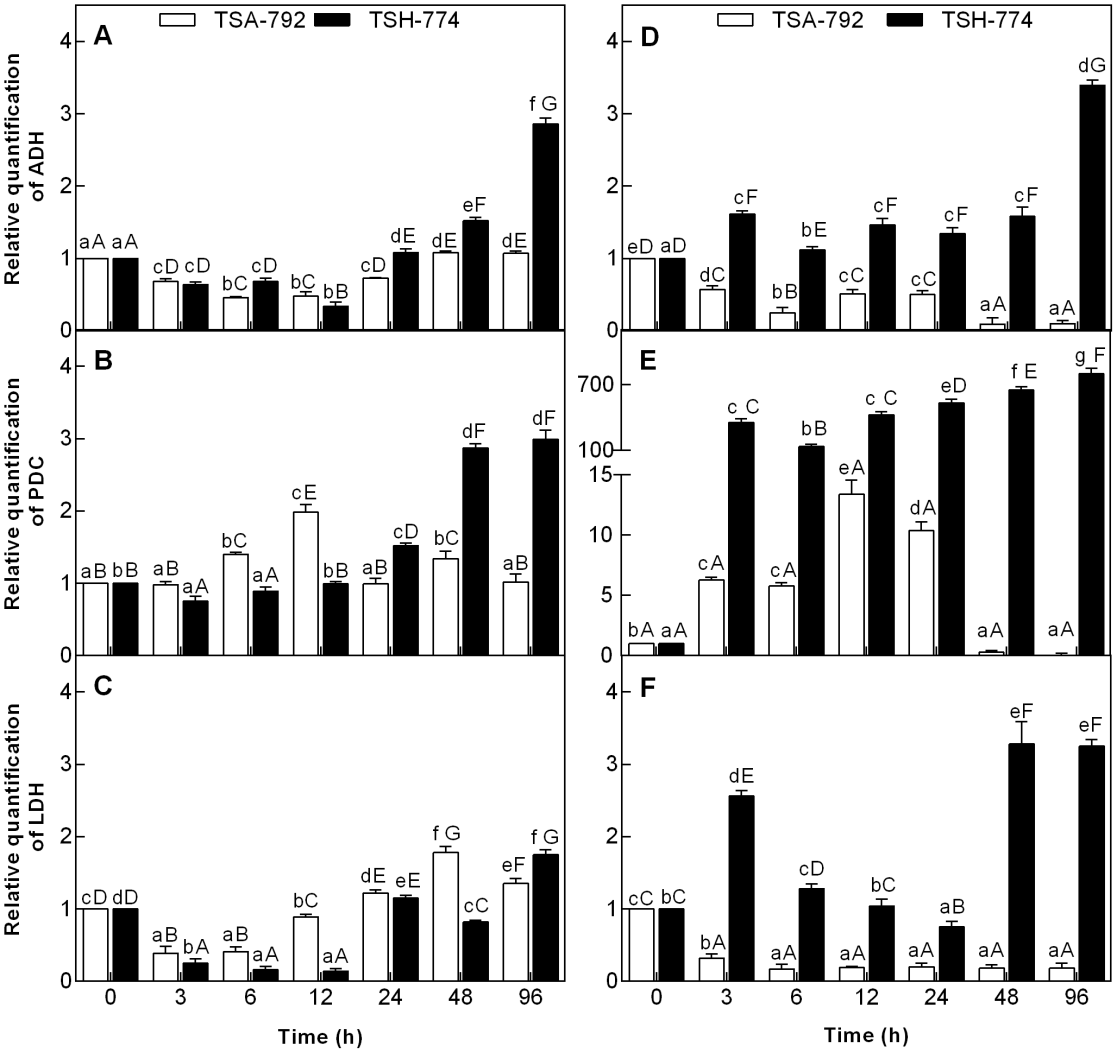
Relative quantity of transcripts encoded by the gene enzymes ADH (A and D), PDC (B and E) and LDH (C and F) quantified by qPCR and normalized by β-tubulin transcripts. The expressed values are relative to the responses of control leaves (A, B and C) and roots (D, E and F) as compared with those of flooded plants in two clonal genotypes of *Theobroma cacao*, (TSA-792 and 774-TSH). The columns represent the mean values ± SE (standard error) of six biological replicates. *Uppercase letters* compare differences between genotypes and *lowercase letters* compare differences within genotypes. Mean comparisons were using a Tukey test (P<0.05).

Soil flooding was observed to induce changes in the pattern of gene expression of enzymes ADH, PDC and LDH in leaves and roots of two clonal genotypes of *T. cacao* (Fig. 1). There has been a decrease in the gene expression of ADH enzyme in leaves of two clonal genotypes of cocoa in the first 12 h of soil flooding; in turn, after 24 h, there has been an increase in the expression of this gene, which was observed to be greater for the TSH-774 clonal genotype (Fig. 1A). Regarding the gene of PDC enzyme in leaves, there was an increase in expression during the first 12 h of soil flooding, followed by a decrease for the TSA-792 clonal genotype after 24 h; in the initial periods of stress, the TSH-774 clonal genotype showed a decrease in the expression of this gene and a high increase of expression 24 h after soil flooding (Fig. 1B). In both clonal genotypes of *T. cacao*, there was a decrease in the gene expression of LDH enzyme after stress induction for 6 h (Fig. 1C). The TSA-792 clonal genotype showed increased expression of this gene after 12 h of soil flooding, whereas the increase observed for TSH-774 has only occurred after 24 h.

Roots of the TSA-792 clonal genotype showed decreased gene expression of ADH enzyme 3 h after soil flooding, whereas the TSH-774 clonal genotype showed increased expression after the same period, and high expression after 96 h of stress induction (Fig. 1D). There has been an increase in the gene expression of PDC enzyme in the roots of two clonal genotypes of *T. cacao* after 3 hours of soil flooding; the TSH-774 genotype showed the highest expression (Fig. 1E). The TSA-792 clonal genotype showed decreased expression of this gene after 48 h of soil flooding, whereas the expression of TSH-774 clonal genotype remained high throughout the period of stress induction (Fig. 1E). A decreased gene expression of LDH enzyme was observed for the roots of TSA-792 clonal genotype after 3 h of soil flooding, which was constant until the end of the experiment (Fig. 1F). On the other hand, the TSH-774 clonal genotype showed a high expression at 3 h of soil flooding, followed by a decrease within 24 hours, and another increase in expression after 48 h of stress induction (Fig. 1F).

### Activity of enzymes ADH, PDC and LDH to soil flooding

**Figure 2:**
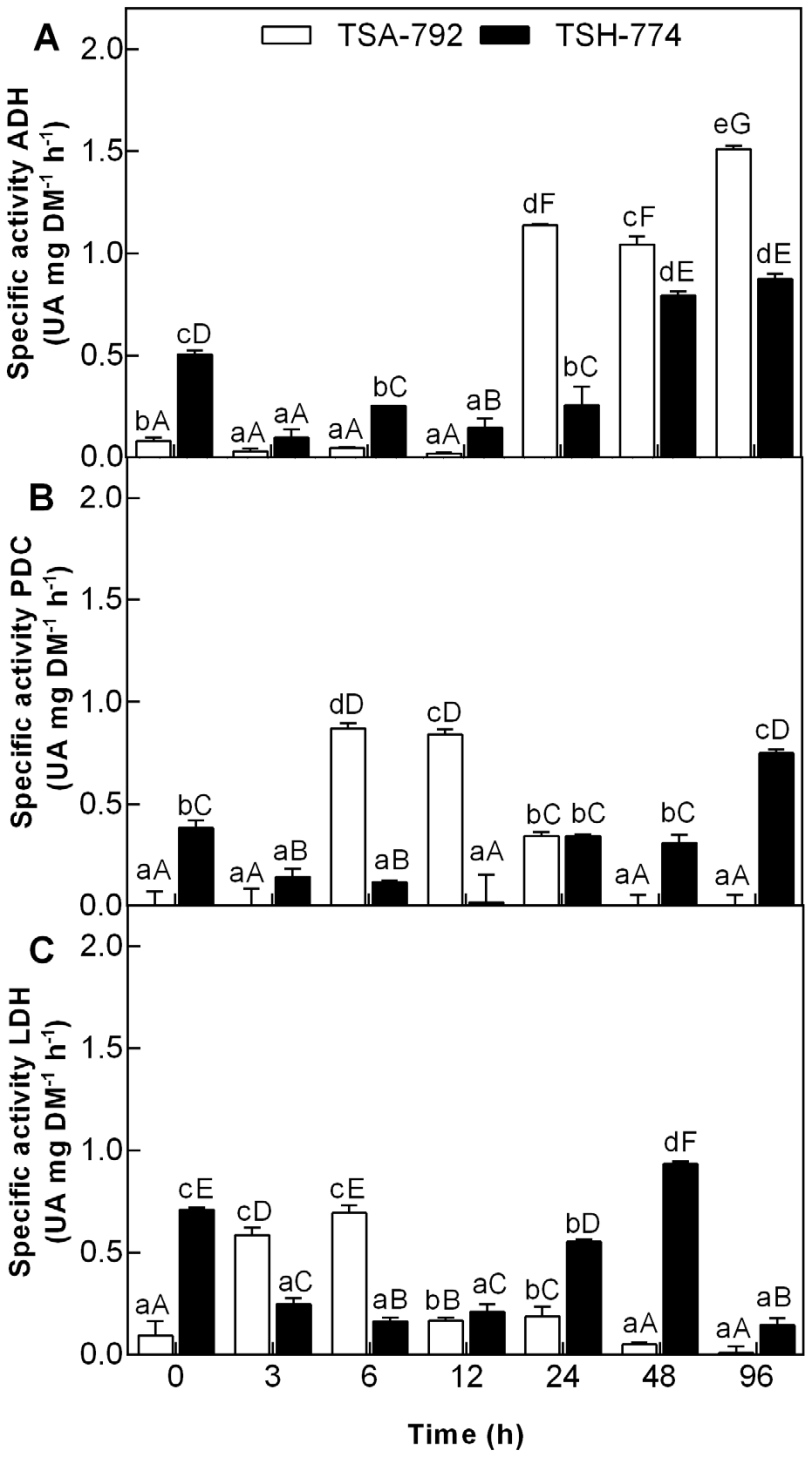
ADH activity (A), PDC (B) and LDH (C) in leaves of plants of TSA-792 and TSH-774 clonal genotypes of *Theobroma cacao* subjected to soil flooding during periods of 0, 3, 6, 12, 24, 48 and 96 h. Mean values of four replicates ± SE (standard error). Lowercase letters compare between genotypes. *Uppercase letters* compare differences between genotypes and *lowercase letters* compare differences within genotypes. Mean comparisons were using a Tukey test (P<0.05). UA - absorbance units.

**Figure 3:**
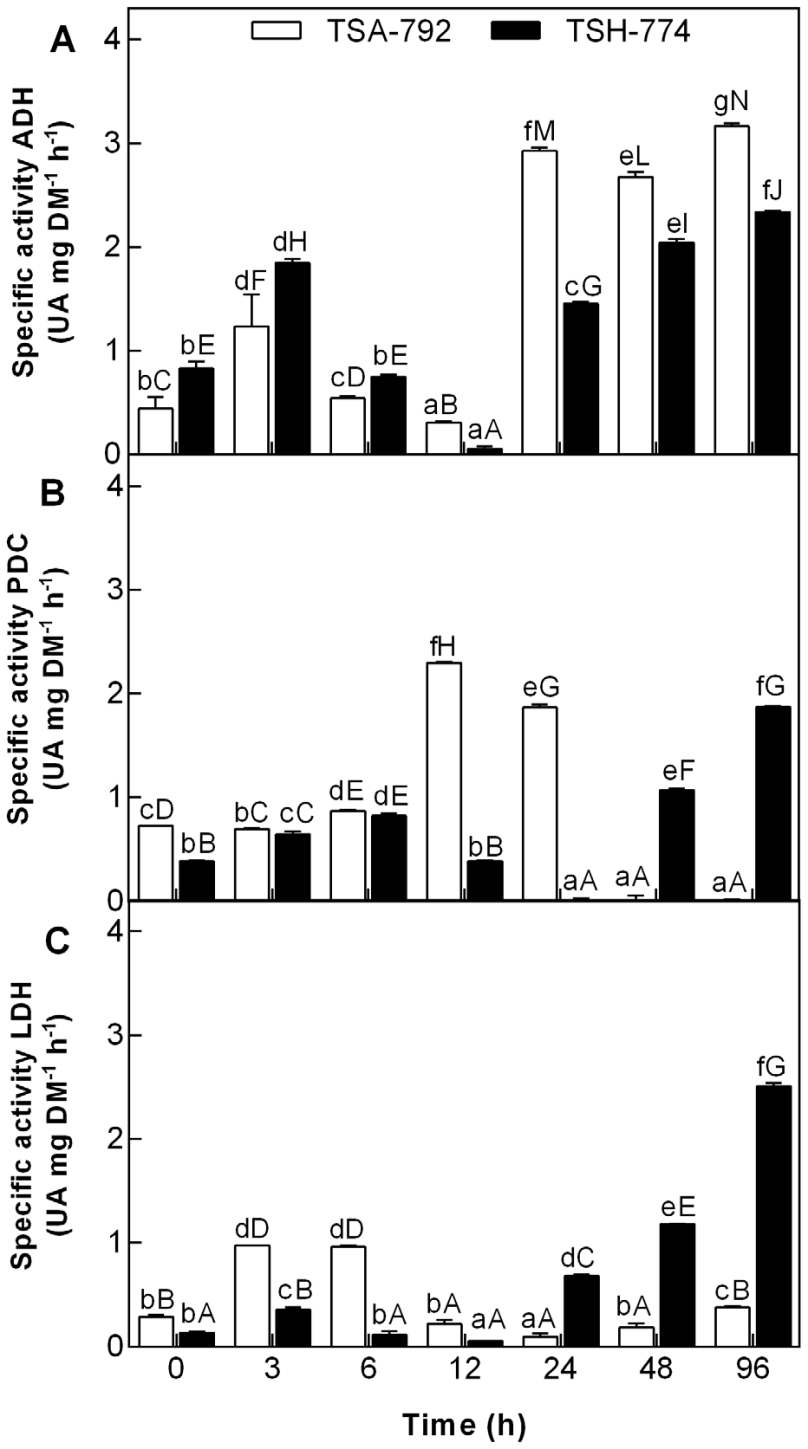
ADH activity (A), PDC (B) and LDH (C) in roots of plants of TSA-792 and TSH-774 clonal genotypes of *Theobroma cacao* subjected to soil flooding during periods of 0, 3, 6, 12, 24, 48 and 96 h. Lowercase letters compare between genotypes. *Uppercase letters* compare differences between genotypes and *lowercase letters* compare differences within genotypes. Mean comparisons were using a Tukey test (P<0.05). UA - absorbance units.

The study of the activity of enzymes involved in anaerobic metabolism of leaves and roots showed significant (P <0.05) differences between genotypes TSA-792 clone TSH-774 and subjected to soil flooding (Fig. 2 and 3). In leaves of both genotypes, ADH activity was low until 12 h of soil flooding, with a significant increase after 24 h, being more pronounced for the TSA-792 clonal genotype (Fig. 2A). The PDC activity was higher in leaves of the TSA-792 between 6 and 12 h, followed by a decrease after 24 h of soil flooding; after this period, the TSH-774 clonal genotype showed increased enzyme activity (Fig. 2B). Regarding LDH activity, increased activity was observed in the leaves of TSA-792 clonal genotype between 3 and 6 h of soil flooding, followed by a decrease after 12 h, which persisted until the end of the experiment; on the other hand, the TSH-774 clonal genotype showed an initial decrease in enzyme activity, followed by a significant increase in the period between 24 and 48 h of stress, and a sharp decrease in the end (Fig. 2C).

Roots of TSA-792 and TSH-774 clonal genotypes showed increased ADH activity at 3 h of soil flooding, followed by a considerable decrease in periods of 6 and 12 h. After 24 h of stress induction, ADH activity was observed to increase and was significantly higher for the TSA-792 clonal genotype (Fig. 3A). At the initial period of soil flooding, PDC activity was similar in the roots of both genotypes, but it showed a significant increase in the roots of TSA-792 clonal genotype after 12 h of stress induction, and decreased after 48 h (Fig. 3B). In this same period, a significant increase in PDC activity was observed for the TSH-774 genotype (Fig. 3B). An increase in LDH activity was observed in the roots of TSA-792 clonal genotype in the period between 3 and 6 h of soil flooding, followed by a rapid decrease after 12 h, which remained until the end of the experiment (Fig. 3C); in turn, the roots of TSH-774 clonal genotypes showed a gradual increase in LDH activity after 24 h of soil flooding (Fig. 3C).

### Profiles of proteins in *T. cacao* to soil flooding

**Figure 4:**
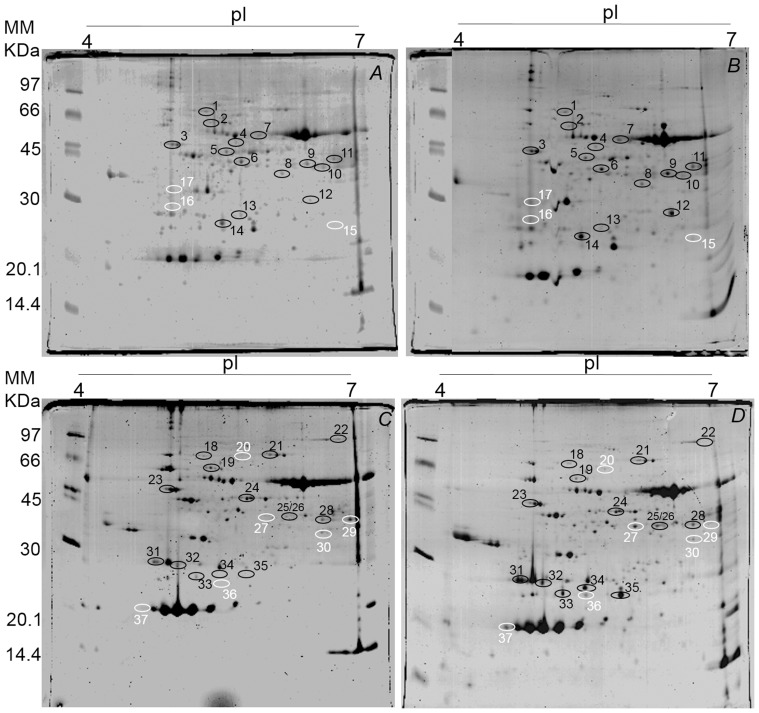
Two-dimensional gel of proteins from leaves of clonal genotypes of *Theobroma cacao* subjected to 96 h of soil flooding. Control (A) and flooded (B) TSA-792, and (C) control and (D) flooded TSH-774. Each gel was loaded with 350 µg of total protein and stained with 0.08% w/v colloidal Coomassie G-250. Black circles indicate spots differentially expressed between control and flooded treatments, whereas white circles indicate spots exclusive to one of the treatments in response to soil flooding.

**Figure 5:**
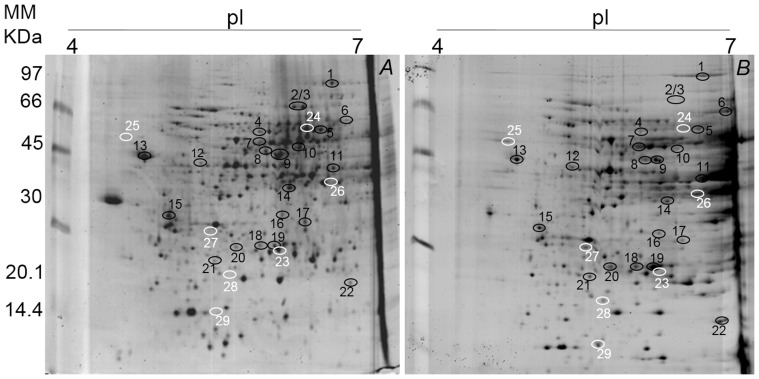
Two-dimensional gel of proteins from roots of the TSA-792 clonal genotype of *Theobroma cacao* subjected to 96 h of soil flooding. *A -* control and *B -* flooded. Each gel was loaded with 350 g of total protein and stained with 0.08% w/v colloidal Coomassie G-250. Black circles indicate spots differentially expressed between control and flooded treatments, whereas white circles indicate spots exclusive to one of the treatments in response to soil flooding.

**Figure 6:**
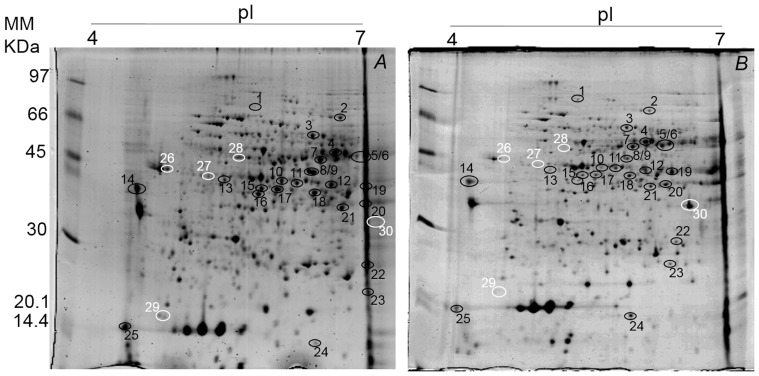
Two-dimensional gel of proteins from roots of the TSH-774 clonal genotype of *Theobroma cacao* subjected to 96 h of soil flooding. *A -* control and *B -* flooded. Each gel was loaded with 350 µg of total protein and stained with 0.08% w/v colloidal Coomassie G-250. Black circles indicate spots differentially expressed between control and flooded treatments, whereas white circles indicate spots exclusive to one of the treatments in response to soil flooding.

A total of 112 and 118 protein spots were identified in the leaves of clonal genotype TSA-792, and 101 and 155 protein spots in the leaves of clonal genotype TSH-774, in the control and flooded treatments, respectively (Fig. 4). Already in the roots 291 and 261 protein spots were identified in clonal genotype TSA-792, and 273 and 239 protein spots in clonal genotype TSH-774, in the control and flooded treatments, respectively (Figs. 5 and 6). A comparative proteomic analysis was employed to investigate the profiles of proteins under flooding-induced stress in clonal genotypes of *T. cacao* (TSA-792 and TSH-774) in order to distinguish levels of stress responses. The analysis has identified 17 and 20 differentially expressed proteins in the control and flooded treatments, in leaves of clonal genotypes TSA-792 and TSH-774, respectively, subjected to 96 h of soil flooding (Fig. 4, Table 1). In contrast, 33 and 31 differentially expressed proteins have been identified in roots, when comparing the control and flooded treatments as for the clonal genotypes TSA-792 e TSH-774, respectively (Figs. 5 and 6; Tables 2 and 3).

**Table 1:** Proteins from leaves of TSA-792 and TSH-774 clonal genotypes of *Theobroma cacao* identified by mass spectrometry.

Spot n°^a^	Accession n°	Protein Name	Theoretical MW(KDa)/pI	Biological process	Fold change ^b^
	**Upregulated**				
1	ACJ11741.1	Heat Shock	70.94/4.94	Response to stress	↑2.0
2	XP_002523404.1	Tcp-1 Cpn60 Chaperonin Family Protein	63.86/5.71	Protein refolding/Cell death	↑2.0
3	ADD60244.1	Ribulose Bisphosphate CarboxylaseOxygenase Activase Chloroplast	51.9/5.0	Defense response to bacterium/Response to light stimulus	↑2.0
4	P41379.1	Dead-Box RNA Helicase-Like Protein	52.7/6.13	Translational initiation	↑2.0
5	XP_002513353.1	Phosphoglycerate Kinase	48.43/5.97	Reductive pentose-phosphate cycle/Glycolysis/Phosphorylation	↑2.5
6	XP_002519002.1	Phosphoribulokinase Precursor	45.47/6.23	Carbohydrate metabolic process Phosphorylation	↑2.5
7	ABW21688.1	Enolase	46.9/5.77	Glycolysis	↑2.2
8	XP_002525379.1	Alcohol Dehydrogenase, Putative	41.01/7.09	Cellular metabolic process	↑2.5
9	XP_002531508.1	Plastidic Aldolase	42.69/8.34	Glycolysis	↑2.0
10	XP_002524262.1	Mitochondrial NAD-Dependent Malate Dehydrogenase	42.69/7.01	Malate metabolic process/Cellular carbohydratemetabolic process	↑3.1
11	CAH60893.1	Formate Dehydrogenase	43.65/7.11	Oxidation reduction	↑2.1
12	ABZ01817.1	Alcohol Dehydrogenase	39.99/7.21	Cellular metabolic process	↑5.9
14	XP_002521576.1	Oxygen-Evolving Enhancer Protein Chloroplast	30.38/6.53	Photosynthesis/Oxidation reduction	↑3.5
18	ACJ11741.1	Heat Shock Protein 70	70.94/4.94	Response to stress	↑2.0
23	ADD60244.1	Ribulose Bisphosphate CarboxylaseOxygenase Activase Chloroplast	51.9/4.99	Defense response to bacterium/Response to light stimulus	↑2.0
24	ABQ41114.1	Monodehydroascorbate Reductase	46.92	Cell redox homeostasis/Electron transport chain	↑2.0
26	ABR13881.1	Arginase	36.97/6.08	Arginine catabolic process/Defense response to bacterium	↑3.7
27	XP_002525379.1	Alcohol Dehydrogenase, Putative	41.01/9.29	Cellular metabolic process	↑2.8
28	XP_002531508.1	Plastidic Aldolase	42.69/8.34	Glycolysis	↑2.5
31	ACO51067.1	Light Harvesting Chlorophyll a b-Binding Protein	28.05/4.95	Photosynthesis, light harvesting/Protein-chromophore linkage	↑4.7
32	XP_002531690.1	Light-Harvesting Complex II Protein Lhcb2	28.48/5.12	Photosynthesis, light harvesting/Protein-chromophore linkage	↑5.0
	**Upregulated**				
33	2WSC_3	Light-Harvesting Complex II Protein Lhcb3	29.73/6.63	Photosynthesis, light harvesting/Protein-chromophore linkage	↑5.2
34	2WSC_3	Light-Harvesting Complex I Chlorophylla b Binding Protein 3	29.73/7.63	Photosynthesis, light harvesting/Protein-chromophore linkage	↑10
35	XP_002521576.1	Oxygen-Evolving Enhancer Protein Chloroplast	28.31/8.75	Photosynthesis/Oxidation reduction	↑15
	**Downregulated**				
13	XP_002519950.1	Chlorophyll a b-Binding	29.92/5.38	Photosynthesis, light harvesting	↓2.5
19	XP_002523404.1	Tcp-1 Cpn60 Chaperonin Family Protein	63.86/5.71	Protein refolding/Cell death	↓2.3
21	XP_002511690.1	Transketolase	78.94/6.34	Energy reserve metabolic process	↓2.0
22	ABO61734.1	Glycine Dehydrogenase	53.12/6.23	Glycine catabolic process	↓3.1
	**Unique to one of**	**the treatments**			
15	ABK81651.1	Glutathione S-Transferase	29.93/8.23	Response to stimulus	N
16	ACO51067.1	Light Harvesting Chlorophyll a b -Binding Protein	28.05/4.95	Photosynthesis, light harvesting/Protein-chromophore linkage	N
17	ABB02412.1	Chloroplast Lipocalin	38.24/5.73	Response to oxidative stress	N
20	AAC17840.1	Vacuolar H^+^-ATPase Catalytic Subunit	43.05/5.08	ATP synthesis/ATP catabolic process	N
25	XP_002525379.1	Alcohol Dehydrogenase, Putative	41.01/9.29	Cellular metabolic process	N
29	XP_002512790.1	Glyceraldehyde-3-Phosphate Dehydrogenase (NADP^+^)	42.69/5.26	Reductive pentose-phosphate cycle/Glycolysis	N
30	XP_002513006.1	NADPH Dependent Mannose 6-Phosphate Reductase	34.94/5.99	Oxidation reduction	N
36	XP_002521576.1	23 KDa Polypeptide Of The Oxygen EvolvingComplex Of Photosystem II	23.71/5.52	Photosynthesis/Oxidation reduction	N
37	AAV41231.1	Trypsin Inhibitor	23.97/4.22	Negative regulation of endopeptidase activity	N

a Number of spots correspond to the numbers in pictures 2-D gel in Fig. 4. ^b^ Proteins differentially expressed in leaves of the genotypes of *T. cacao* TSA-792 and TSH-774 subjected to 96 h of flooding. Proteins with abundance increased (↑) or decrease (↓) or (N) unique to one of the treatments (control and flooding) in response to flooding. The number represents changes in the rate of protein abundance in the treatment compared to control flooded. e-value = 0.

**Table 2:** Proteins from roots of the TSA-792 genotype of *Theobroma cacao* identified by mass spectrometry.

Spot n°^a^	Accession n°	Protein Name	Theoretical MW(KDa)/pI	Biological process	Fold change ^b^
	**Upregulated**				
1	XP_002513404.1	Eukaryotic Translation Elongation Factor	99.23/5.79	Protein biosynthesis	↑2.0
6	AAF04851.1	Alcohol Dehydrogenase A	41.64/6.57	Cellular metabolic process	↑7.5
7	ABW21688.1	Enolase	47.94/5.48	Glycolysis	↑3.3
	ACJ11711.1	UDP-Glucose Pyrophosphorylase	51.28/5.51	Metabolic process	↑3.3
11	AAF04851.1	Alcohol Dehydrogenase A	41.64/6.57	Cellular metabolic process	↑9.5
	XP_002528593.1	Fructose Bisphosphate Aldolase	40.92/6.53	Pentose-phosphate shunt/Glycolysis	↑9.5
12	XP_002519823.1	Acetylornithine Deacetylase	52.74/5.18	Proteolysis	↑2.0
13	AAL92037.1	Peroxidase	37.15/4.43	Response to oxidative stress	↑2.1
18	ABA46792.1	Triosephosphate Isomerase	27.21/5.4	Pentose-phosphate shunt/Glycolysis/Gluconeogenesis	↑3.0
19	ABR68691.2	Ascorbate Peroxidase	27.56/5.42	Response to oxidative stress	↑2.9
20	XP_002513374.1	Proteasome Subunit Alpha Type-2-B	25.64/5.34	Defense response to bacterium/Ubiquitin-dependent protein catabolic process	↑2.4
21	XP_002530662.1	3-Isopropylmalate Small Subunit	26.81/5.69	Leucine biosynthetic process	↑2.3
22	AAX86687.1	Nonsymbiotic Hemoglobin	18.35/9.07	Oxygen transport/Nitrogen fixation/Response to stress	↑5.3
	**Downregulated**				
2	XP_002511690.1	Transketolase	80.61/6.34	Energy reserve metabolic process	↓6.4
3	YP_004237280.1	ATPase Subunit 1	55.26/5.74	ATP synthesis	↓6.5
4	NP_565040.2	Alanine Aminotransferase	59.27/5.97	Response to hypoxia/biosynthetic process1-aminocyclopropane-1-carboxylate	↓2.1
5	AAC67586.1	Pyrophosphate-DependentPhosphofructokinase Beta Subunit	61.63/5.8	Glycolysis/Phosphorylation	↓2.9
8	XP_002522953.1	DNA Binding Protein	45.83/5.65	Nucleotide binding	↓2.9
	NP_001235426.1	Mitogen-Activated Protein Kinase	48.97/5.51	Protein amino acid phosphorylation/Cell division	↓2.9
9	XP_002519309.1	S-Adenosyl methionine Synthetase	42.97/5.5	One-carbon metabolic process	↓3.5
10	XP_002512941.1	Diaminopimelate Decarboxylase	58.76/7.05	Lysine biosynthetic process via diaminopimelate	↓3.3
14	ADD51354.1	Anthocyanidin Reductase	36.27/6.05	Flavonoid biosynthetic process	↓8.0
	ACJ11738.1	Cytosolic Malate Dehydrogenase	36.57/6.67	Cellular carbohydrate metabolic process	↓8.0
15	CAC86003.1	Aspartic Proteinase	59.75/4.86	Proteolysis/Lipid metabolic process	↓2.0
16	ACJ11750.1	Lactoylglutathione Lyase-Like Protein	32.5/5.64	Carbohydrate metabolic process	↓3.3
17	BAJ10727.1	Protein Transport Protein Sec13	32.71/5.65	Membrane budding	↓2.7
	**Unique to one of**	**the treatments**			
23	AAD11255.1	Class I Chitinase	34.84/5.06	Chitin catabolic process/Defense response/Cell wall	N
24	YP_004237280.1	ATP Synthase F1 Subunit 1	55.26/5.74	ATP synthesis	N
25	XP_002533858.1	26S Proteasome Non-ATPase Regulatory	44.54/4.2	Protein catabolic Process proteolysis	N
26	ACJ11728.1	G lyceraldehyde-3-PhosphateDehydrogenase	36.67/8.12	Reductive pentose-phosphate cycle/Glycolysis	N
27	XP_002525377.1	Carbonic Anhydrase	28.07/5.16	Carbon utilization	N
28	XP_002523700.1	Glyoxalase I	26.8/8.42	Response to stress	N
29	AAV41231.1	Trypsin Inhibitor	23.92/5.92	Negative regulation of endopeptidase activity	N

a Number of spots correspond to the numbers in pictures 2-D gel in Fig. 5. ^b^ Proteins differentially expressed in roots of the genotype of T. cacao TSA-792 subjected to 96 h of flooding. Proteins with abundance increased (↑) or decrease (↓) or (N) unique to one of the treatments (control and flooding) in response to flooding. The number represents changes in the rate of protein abundance in the treatment compared to control flooded. e-value = 0.0.

**Table 3:** Proteins from roots of the TSH-774 genotype of *Theobroma cacao* identified by mass spectrometry.

Spot n°^a^	Accession n°	Protein Name	Theoretical MW(KDa)/pI	Biological process	Fold change ^b^
	**Upregulated**				
1	Q42910.1	Phosphate Dikinase 1	98.72/4.95	Photosynthesis	↑2.8
5	ACJ11711.1	UDP-Glucose Pyrophosphory-lase	51.28/5.51	Metabolic process	↑2.1
24	AAQ08194.1	Eukaryotic Translation Initiation Factor 5a2	17.37/5.54	Protein biosynthesis	↑3.4
	**Downregulated**				
2	XP_002511690.1	Transketolase	80.61/6.34	Energy reserve metabolic process	↓2.1
3	XP_002519975.1	Bisphosphoglycerate-IndependentPhosphoglycerate Mutase	61.06/5.52	Glycolysis	↓2.2
4	XP_002522805.1	S-Adenosyl-L-Homocysteine Hydrolase	53.29/5.72	One-carbon metabolic process/Posttranscriptional gene	↓2.4
6	XP_002510911.1	Enolase	46.9/5.77	Glycolysis	↓5.1
7	XP_002517676.1	6-Phosphogluconate Dehydrogenase	54.06/5.65	Pentose-phosphate shunt	↓2.0
8	ACK57683.1	Peroxidase 12	39.03/5.54	Response to oxidative stress	↓3.6
9	XP_002515204.1	S-Adenosyl-methionine Synthetase	43.03/5.53	One-carbon metabolic process	↓4.4
10	ABZ01817.1	Cinnamyl Alcohol Dehydrogenase	39.99/5.3	Cellular metabolic process	↓3.8
11	ABZ01817.1	Cinnamyl Alcohol Dehydrogenase	39.99/5.3	Cellular metabolic process	↓2.8
12	A/AAF04851.1	Alcohol Dehydrogenase	41.64/6.57	Cellular metabolic process	↓2.3
13	1/NP_567017.2	Beta-Hexosaminidase	64.25/5.59	Carbohydrate metabolic process	↓2.1
14	AAC33732.1	Pathogenesis-Related Protein 4b	29.42/3.99	Defense response/Response tobiotic stimulus	↓3.0
15	ACT32029.1	Caffeic Acid O-Methyltransferase	40.02/5.18	Lignin biosynthetic process/Methylation	↓2.6
16	ABM64799.1	Flavanone 3-Hydroxylase	40.96/5.08	Flavonoid biosynthetic process	↓7.2
17	XP_002531678.1	Adenosine Kinase	37.32/5.05	Adenosine salvage/Phosphorylation	↓3.0
18	ACT32029.1	Caffeic Acid O-Methyltransferase	40.02/5.18	Lignin biosynthetic process/Methylation	↓2.3
19	A/AAF04851.1	Alcohol Dehydrogenase	41.64/6.57	Cellular metabolic process	↓3.7
20	XP_002528593.1	Fructose Bisphosphate Aldolase	40.92/6.53	Pentose-phosphate shunt/Glycolysis	↓3.2
21	ACJ11738.1	Cytosolic Malate Dehydrogenase	35.7/6.67	Cellular carbohydrate metabolic process	↓3.0
22	ABA46792.1	Triosephosphate Isomerase	27.21/5.4	Pentose-phosphate shunt/Glycolysis	↓7.1
23	ABX79342.1	Superoxide Dismutase	25.69/7.6	Removal of superoxide radicals	↓3.7
25	AAV41231.1	Trypsin Inhibitor	23.92/5.92	Negative regulation of endopeptidase activity	↓5.0
	**Unique to one of**	**the treatments**			
26	AAL92037.1	Peroxidase	37.15/4.43	Response to oxidative stress	N
27	ABB17002.1	Transaldolase	47.79/5.47	Pentose-phosphate shunt	N
28	XP_002533858.1	26S Proteasome Non-ATPaseRegulatory	53.31/4.8	Protein catabolic Process proteolysis	N
29	AAV41231.1	Trypsin Inhibitor	23.92/5.92	Negative regulation of endopeptidase activity	N
30	AAR13288.1	Annexin 2	35.86/6.22	Calcium-dependent phospholipid binding	N

a Number of spots correspond to the numbers in pictures 2-D gel in Fig. 6. ^b^ Proteins differentially expressed in roots of the genotype of *T. cacao* TSH-774 subjected to 96 h of flooding. Proteins with abundance increased (↑) or decrease (↓) or (N) unique to one of the treatments (control and flooding) in response to flooding. The number represents changes in the rate of protein abundance in the treatment compared to control flooded. e-value = 0.0.

Among the 17 proteins identified in leaves of the TSA-792 clonal genotype, 3 spots were exclusive to the flooded treatment (spots 15, 16 and 17), 13 spots showed increased expression, whereas one showed decreased expression (Figs. 4A and B). Regarding the 20 proteins identified in leaves of TSH-774 clonal genotypes, 11 spots increased and three decreased their expression; in addition, 6 spots were exclusive, whereof one (spot 29) was exclusive to the control treatment and 5 (spots 20, 25, 30, 36 and 37) were exclusive to the flooded treatment (Figs. 4C and D). The proteins identified in both genotypes are listed in Table 1.

Regarding the differentially expressed proteins found in the roots of TSA-792 clonal genotype, 10 spots were observed to have an increased expression, whereas 12 showed a decreased expression; 7 spots were regarded as exclusive, where of 2 pertained to the control (spots 23 and 24) and 5 (spots 25, 26, 27, 28 and 29) pertained to the flooded treatment (Fig. 5). The identified proteins are described in Table 2. On the other hand, 5 exclusive spots were observed for the TSH-774 clonal genotype: 4 in the control treatment (spots 26, 27, 28 and 29) and 1 in the flooded control (spot 30); 3 spots showed increased expression and 20 spots showed decreased expression (Fig. 6). The identified proteins are listed in Table 3.

## Discussion

Bertolde et al. (2012) observed in plants of clonal genotypes of *T. cacao*, TSH-774, in response to flooding the nonstomatal limitations to photosynthesis, where the decrease in Fv/Fm values indicated possible damage to the PSII light-harvesting complex; oxidative stress; increase leaf chlorosis; decreased roots carbohydrates concentrations, resulting in death of several plants after 30 d of flooding. The induction of responses associated with anaerobic metabolism in plants of clonal genotypes of *T. cacao* (TSH and TSA-792-774) to anoxia was verified by determining the gene expression of ADH, PDC and LDH enzymes in leaves and roots (Fig. 1). These genes have been described in the literature as being expressed in anoxia conditions [25], [26], [27], [28], [29]. High levels of transcripts of *adh, ldh* and *pdc* were observed in leaves and roots of TSH-774 clonal genotypes subjected to soil flooding as compared with the TSA-792 clonal genotype (Figs. 1). Yet, the induction of genes encoding enzymes of glycolytic or fermentative routes is not directly correlated with the ability to survive under conditions of O_2_ deficiency, as not all come to be translated [15]. Ellis et al. (2000) have found that the increased expression of *adh* and *pdc* in *Gossypium hirsutum* has not promoted significant increases in survival rates under waterlogged soil conditions. Generally, the expression patterns of *adh*, *pdc* and *ldh* were distinguished considering the organs and evaluation periods for the two clonal genotypes of *T. cacao* (Fig. 1).

The induction of enzyme genes of fermentation seems not to be coordinated among the different plant organs, with peaks of transcripts at different times of anoxia, as verified for *Acorus calamus* and *Oryza sativa* [30], [31]. The role of anaerobic respiration as an adaptive pathway for the survival of plants in flooded environments has been demonstrated in several studies [14], [32], [33]. This study has assessed the activities of ADH, PDC and LDH in leaves and roots of clonal genotypes of *T. cacao*, TSA-792 and TSH-774. As observed in both genotypes (Figs. 2 and 3), the increased activity of these enzymes in waterlogged conditions suggests that there have been changes in the metabolic activities, from aerobic respiration to fermentative pathways; that was critical to maintain sufficient levels of ATP and sustain plant growth [34]. O_2_ deficiency has been associated with increased ADH, PDC and LDH activity in *Glycera maxima, Senecio aquaticus, O. sativa* and *Vigna radiate* [14], [32], [35].

The significant increase in ADH activity in leaves and roots of both genotypes assessed after 24 h of anoxia (Figs. 2A and 3A) was similar in *Cyperus rotundus* [33]. Despite there has been increased ADH activity in both genotypes, such increase was considerably higher in the TSA-792 clonal genotype in leaves and roots. Flood-tolerant plants show the highest increases in ADH activity under anoxia conditions, as compared with susceptible plants; that was observed in a study involving contrasting genotypes of *Vigna radiate* for flooding tolerance [14], and confirmed in this study. The increased ADH activity in roots of TSA-792 clonal genotype (Fig. 3A) was not followed by an increase in the number of transcripts of such enzyme (Fig. 1D), thus suggesting the possible activation of preexisting enzymes, without necessarily increase the number of transcripts.

In alcoholic fermentation, PDC serves as a limiting factor under oxygen-deficient conditions [36]. In this study, an increase in PDC in leaves and roots of TSA-792 clonal genotype has been observed in the periods from 6 to 12 h and 12 to 24 h, respectively (Figs. 2B and 3B). This period precedes the significant increase verified in the ADH activity of this genotype, suggesting the initial activation of PDC with conversion of pyruvate into acetaldehyde and subsequent activation of ADH with the conversion of acetaldehyde into ethanol (Figs. 2A and 3A). Yet, the increased PDC activity in TSH-774 clonal genotype was observed after 24 h in leaves, and after 48 h in roots (Figs. 2B and 3B); such period was similar to that of ADH activation in this genotype. Likewise in this study, have demonstrated that, in *O. sativa* plants, the transfer into anoxia promotes increased PDC and ADH activities, while the PDC activity is always lower than that of ADH (Figs. 2B and 3B). The qPCR data indicating overexpression of the corresponding gene of PDC enzyme (Fig. 2B) and its low enzyme activity (Fig. 3B) in the roots of TSH-774 clonal genotypes suggest an attempt by the plant to compensate the low enzyme activity by increasing the number of transcripts. This enzymes are allosterically regulated as reflected by sigmoid steady state kinetics and lag phases in their progress curves. Higher oligomers (octamers) have been described for PDCs from plant seeds [37]. The substrate piruvate activates the initially inactive PDCs in a time-dependent manner, covalently bound substrate at the regulatory site of PDC triggers allosteric enzyme activation. However, studies on structure function relationships of yeast PDCs showed that the dimer is the minimum functional unit of the enzyme displaying considerable catalytic activity [38]. Variants of the enzyme from *T. cacao* genotypes may respond differentially to this mechanism as occur in yeast [38].

In regard to LDH activity, increased activity was only observed in leaves and roots of TSA-792 clonal genotype in the initial period of stress (3 and 6 h), whereas such increase was only noted in the same organs of TSH-774 clonal genotypes after 24 h of stress induction (Figs. 2C and 3C). The responses of plants to anoxia are initially characterized by the production of lactic acid, which causes cellular acidosis, this acidosis promotes the activation of PDC and leads to LDH inhibition, causing a switch from latic fermentation to alcoholic fermentation [39]. The accumulation of lactic acid in prolonged periods of soil flooding increases cellular acidosis, causes cell death and increases the susceptibility to O_2_ deficiency [40]. Regulation or inhibition of LDH activity under anoxia could, therefore, prevent cellular acidosis [41]. The TSA-792 clonal genotype is seemingly more efficient in regulating the formation of lactic acid when subjected to O_2_ deficiency conditions as compared with the TSH-774 clonal genotype (Figs. 2C and 3C), preventing cellular acidosis. The regulation of lactic fermentation in the leaves of TSA-792 clonal genotype has probably occurred through translation, or even by enzyme inactivation, since there was an increase in the gene expression of LDH enzyme (Fig. 1C), without a sub-sequential increase in enzyme activity (Fig. 2C). ADH activity, in turn, was considerably higher than that of LDH in the two assessed genotypes, in flooded soil conditions, hence indicating that alcoholic fermentation is probably the dominant pathway under anaerobic conditions.

Transcriptome and proteome analyses have identified numerous genes that are expressed in response to oxygen deficiency-induced stress in plants [17], [19], [20], [21], [40], providing evidence of the complexity of plant responses to such stress. Besides from glycolytic proteins and fermentation enzymes, this study has identified proteins associated with photosynthesis, protein metabolism, oxidative stress, among others.

Photosynthesis-associated proteins like the spots 14, 16, 17, 31–36 (Table 1) showed increased expression under conditions of stress by anoxia in the two clonal genotypes of *T. cacao*, suggesting a possible attempt to maintain the photosynthetic rate, which shows decreases in such conditions [23], [24].

The main consequence of soil flooding is O_2_ deficiency in the rhizosphere. Thus, fermentation predominates in ATP formation and NAD^+^ regeneration, increasing glycolytic flux in plants. In this study, leaves and roots of TSA-792 and TSH-774 clonal genotypes showed changes in the expression of proteins involved in carbon metabolism and energy production in response to stress. In the TSA-792 clonal genotype, there was an increased expression of proteins involved in glycolysis, such as phosphoglycerate kinase (spot 5), enolase (spot 7) and plastidic aldolase (spot 9) in leaves, with increases of the order of 2.5, 2.2 and 2.0 times, respectively (Fig. 4, Table 1); e. enolase and UDP-glucose pyrophosphorylase (UGPase) (spot 7), fructose-bisphosphate aldolase (spot 11), triosephosphate isomerase (spot 18), with increases of the order of 3.3, 9.5 and 3.0 times, respectively; and the increased expression of dehydrogenase and glyceraldehyde-3-phosphate (spot 26) was exclusive of the flooded treatment (Fig. 5, Table 2).

On the other hand, in leaves of TSH-774 clonal genotype, there was an increase of the order of 2.5 times in the expression of plastidic aldolase (spot 28) (Fig. 4, Table 1). However, there were decreases in the expression of glycolytic proteins in roots, such as bisphosphoglycerate-independent phosphoglycerate mutase (spot 2), enolase (spot 6), fructose-bisphosphate aldolase (spot 20) and triosephosphate isomerase (spot 22) of the order of 2.2, 5.1, 3.2 and 7.1 times, respectively; a single increase of the order of 2.1 times was observed in the expression of UGP-ase (spot 5) (Fig. 6, Table 3).

The phosphoglycerate kinase enzyme catalyzes the formation of 3-phosphoglycerate from 1.3-bisphosphoglycerate and generates ATP by transferring a phosphate group to ADP in this reaction. The reaction products are strongly favored by the presence of unstable high-energy 1.3-bisphosphoglycerate [12]. Enolase, an essential enzyme in glycolysis, catalyzes the reversible dehydration of 2-phosphoglycerate into phosphoenolpyruvate. The phosphate bound to the 2 position of pyruvate also has a high negative free energy of hydrolysis, allowing the transfer of the phosphate group to ADP in the subsequent reaction. The anaerobic expression of enolase has been demonstrated in other studies [21], [43]. The induction of glycolytic enzymes during anoxia stress therefore contributes to the maintenance of ATP production, as observed for the TSA-792 clonal genotype. The UGPase catalyzes the reversible production of UDP-glucose and pyrophosphate (PPi) from glucose-1-P and UTP. PPi can be used by plants as an alternative energy donor during ATP deficiency [44]. The hydrolysis of PPi provides half the energy released by ATP hydrolysis; yet, unlike what happens to the amount of ATP during anoxia, the PPi content remains relatively stable [45].

Regarding the proteins involved in fermentative pathways, there were two ADH with increases of the order of 2.5 (spot 8) and 5.9 (spot 12) times in the expression in leaves (Fig. 4, Table 1) and 7.5 (spot 6) and 9.5 (spot 11) times in roots of the TSA-792 clonal genotype (Fig. 5, Table 2). Yet, for the TSH-774 clonal genotype, differences in the expression of ADH were observed in leaves and roots. In leaves, two ADH (spots 25 and 27) were identified: one only in the flooded treatment (spot 25) and another (spot 27) with an increase of the order of 2.8 times in the expression (Fig. 4, Table 1). In roots, four ADH were identified, but there were decreases of the order of 3.8 (spot 10), 2.8 (spot 11), 2.3 (spot 12) and 3.7 (spot 19) times in the expression (Fig. 6, Table 3). Pechanova et al. (2010) have identified increased expression of different ADH in leaves and roots of *Populus deltoides*, which is a flood-tolerant tree species; in turn, was identified increased expression of ADH in leaves of *Gossypium hirsutum*, which is a species susceptible to flooding [25].

ADH reduces acetaldehyde to ethanol with a concomitant reoxidation of NAD^+^, which is essential for the continuation of glycolysis. Thus, plants keeps redox status unchanged for a prolonged period, contributing to the survival of plants under oxidative stress [21]. The accumulation of ethanol produced under anoxic conditions only occurs in tree species non-tolerant to stress; tolerant species effectively transport ethanol from roots to leaves, where it is efficiently converted to acetyl-CoA via ADH and used in leaf metabolism [42], [46]. Data on the expression (Figs. 4 and 5) and ADH activity (Figs. 3A and 4A) observed for the flood-tolerant TSA-792 clonal genotype [23] suggest the use of a similar mechanism for survival in oxygen-deficient soils. Unlike the TSA-792 clonal genotype, the TSH-774 genotype susceptible to soil flooding [23] showed a repression in the expression of ADH and proteins involved in glycolysis in roots (Fig. 6) at 96 h of flooding, suggesting loss of ability to maintain energy production for extended periods. The identification of more than one ADH spot indicates probable different isoforms.

Some authors suggest that metabolic pathways involving nitric oxide (NO) and hemoglobin (Hb) provide an alternative type of respiration to mitochondrial electron transport under oxygen deprivation conditions [47]. In this study, only roots of the TSA-792 clonal genotype exhibited a Hb (spot 22) with increased expression of the order of 5.3 times in response to soil flooding (Fig. 5, Table 2). According to [48], the overexpression of Hb confers protection to *Arabidopsis thaliana* plants under severe hypoxic conditions.

There has been a change in the expression pattern of trypsin inhibitors in the assessed genotypes. In leaves of the TSH-774 clonal genotype (spot 37) and roots of the TSA-792 genotype (spot 29), trypsin inhibitors were found to be exclusive in the flooded treatment (Figs. 4 and 5, Table 1 and 3); in turn, one spot exclusive to the control treatment (spot 29) and another with decreased expression of the order of 5.0 times (spot 25) were found in the roots of the TSH-774 clonal genotype (Fig. 6, Table 3). Trypsin inhibitors can regulate the proteolytic activity of proteases in plants [49] in a way that reduced levels of trypsin inhibitors may, therefore, contribute to the degradation of specific proteins that are not necessary during anoxia. This activity can be also performed by the 26S proteasome non-ATPase regulatory subunit (spot 25) induced in roots of the TSA-792 clonal genotype subjected to soil flooding (Fig. 5, Table 2). The aerobic degradation of proteins would be an efficient way to synthesize new proteins by amino acid recycling, instead of using energy-consuming processes such as ion transport, nitrogen assimilation and synthesis of new amino acids [50]. Interestingly, these trypsin inhibitors may also perform other functions such as helping to protect cells against free radicals through the regeneration of ascorbic acid [51], [52]. Thus, the degradation of this protein during anoxia may have implications for oxygen re-entry, causing oxidative stress in the plant.

One of the deleterious effects of anoxia or hypoxia is the accumulation of reactive oxygen species (ROS) [53]. Among the several enzymes that help controlling ROS levels and protecting cells under stress conditions, superoxide dismutase, catalase and peroxidase play an important role. Some authors have noted an increase in peroxidase activity in plants subjected to soil flooding conditions, which was found to be a mechanism for reduction of ROS [54], just as observed in this study involving roots of TSA-792 clonal genotype, with increases of the order of 2.1 and 2.9 times in the expression of peroxidase (spot 13) and ascorbate peroxidase (spot 19), respectively (Fig. 5, Table 2). Yet, studies involving plant species susceptible to soil flooding have indicated a decrease in the expression of peroxidases and superoxide dismutases, resulting in the accumulation of ROS under anoxic stress [21], [22], [55], similar to that verified in roots of TSH-774 clonal genotypes, with decreases of the order of 3.6 and 3.7 times in the expression of peroxidases (spot 8) and superoxide dismutase (spot 23), respectively (Fig. 6, Table 3).

This study has identified a signaling protein - mitogen-activated protein kinase (MAPK) (spot 8) - in the TSA-792 genotype, which showed decreases of the order of 2.9 times in expression in response to soil flooding (Fig. 5, Table 2). According to [49], the induction of MAPK in roots subjected to flooding triggers the activation of a MAPK cascade that plays a role in the regulation of ethylene biosynthesis. Signaling occurs within the first hours of stress induction; the decrease in this protein can be therefore attributed to the period in which the analysis of steady accumulation of proteins was performed (96 h after the start of soil flooding) and the plant developed morphological adaptations to the stress [12]. Another protein that showed decreased expression was the S-adenosylmethionine synthetase (SAM synthetase), 3.5 (spot 9, Fig. 5) and 4.4 (spot 9, Fig. 6) times less expressed in the roots of TSA-792 and TSH-774 clonal genotypes genotypes, respectively, in response to soil flooding. SAM synthetase is a key enzyme in the synthesis of S-adenosyl-L-methionine, which is an important metabolic component in many cellular processes, including the biosynthesis of ethylene. As observed in other studies, the decrease in the expression of such enzyme in both genotypes of *T. cacao* is probably associated with a decrease in ethylene production in roots subjected to long periods of flooding [21], [43].

Ethylene production occurs at the initial period of flooding, and its accumulation leads to cell death, with characteristics similar to those of programmed cell death (PCD) in animal cells [15]. This study has identified proteins related to PCD, such as Tcp-1/Cpn60 chaperonin family (spots 2 and 19, Table 1) expressed in leaves, and eukaryotic translation initiation factor (eIF) (spot 1, Table 2; spot 24, Table 3) expressed in roots [56]. Tcp-1/Cpn60 chaperonin family was 2.0 times more expressed in leaves of the TSA-792 clonal genotype and 2.3 less expressed in leaves of the TSH-774 clonal genotypes, when subjected to soil flooding. On the other hand, eIF was 2.0 and 3.4 times more expressed in roots of the control treatment of TSA-792 and TSH-774 clonal genotypes, respectively. PCD is one of the mechanisms involved in the formation of aerenchyma - tissue consisting of spaces or gaps filled with gas, which extends from the roots to the aerial part of the plant – during soil flooding. This tissue provides the plant an alternative pathway to obtain O_2_, forming an internal aeration system. Moreover, the formation of lysigenous aerenchyma ends up reducing the number of cells that would consume oxygen, thus favoring survival in conditions of low O_2_ availability [21].

In short, results of this study have shown that the tolerance mechanisms of TSA-792 genotype to soil flooding were involved in multiple changes in metabolism, including: (i) induction of glycolytic enzymes, favoring the maintenance of ATP production; (ii) control of key enzymes involved in fermentation, (iii) overexpression of ADH in leaves and roots, suggesting ethanol production in the roots, plus NAD^+^ regeneration and metabolism in the leaves, preventing its accumulation in the cells; (iv) regulation of LDH activity, possibly preventing the accumulation of lactic acid and the resulting cellular acidosis; (v) expression of enzymes involved in controlling the levels of ROS and cell protection. Hence, the ability to maintain glycolysis and induce fermentation was observed to play an important role in anoxic tolerance in cacao genotypes and may also serve to distinguish tolerant and susceptible genotypes.

## Material and Methods

### Plant material and growing conditions

*Theobroma cacao* clonal genotypes contrasting for flooding tolerance (TSA-792 - tolerant and TSH-774 - susceptible) [23], [24], with high productivity and regarded as resistant to witches' broom disease have been evaluated. These two genotypes were provided by the CEPEC/CEPLAC’s Cacao Germplasm Bank of Ilhéus, Bahia (14°47′S, 39°16′W, 55 m asl), Brazil. The genotypes were multiplied through stem cuttings obtained from branch tips of five years-old plagiotropic plants. The rooting of cuttings was performed in 285 cm^3^ conical plastic tubes containing organic substrate (peat and *Pinnus* milled bark+shredded coconut fiber) at a 1∶1 ratio, enriched with macro and micronutrients, following the crop requirements [57]. Five months after rooting, the plants were transplanted to 25 L plastic pots filled with Oxisol, and fertilized according to the nutrient requirements of the species, in a greenhouse at Universidade Estadual de Santa Cruz (UESC), Ilhéus, BA, Brazil. Substrate humidity was maintained near field capacity, for six months. After this period, the flooding treatment was obtained by sealing up the lower end part of 20 pots for each genotype and filling them with water up to 20 mm above ground level, for a period of 40 days. In the control treatment, plants of each genotype remained in pots with the lower end part perforated for drainage of excess irrigation water. Leave and root samples were collected at regular intervals of 0, 3, 6, 12, 24, 48 and 96 h after application of treatments for flooded plants and controls. Plant samples were stored at −80°C after fixation in liquid nitrogen and then lyophilized and stored at −20°C.

### Real-time PCR

RNA extraction from leave and root samples collected 0, 3, 6, 12, 24, 48 and 96 h after treatment application was performed using the RNAqueous kit (Ambion, Life Technologies, Carlsbad, California), according to the manufacturer's instructions. Afterwards, the RNA samples were subjected to electrophoresis on 1% agarose gel so as to assess whether RNA was intact and, therefore, able to be subjected to the first-strand synthesis reaction. The RNA samples were used to synthesize cDNA with Revertaid H-Minus Reverse Transcriptase (Fermentas, Thermo Fisher Scientific Inc., Waltham, MA), according to the manufacturer's instructions, using oligo d(T)15 primers. The reactions were incubated at 65°C, for 5 min., 37°C, for 5 min., 42°C, for 60 min. and 70°C, for 10 min.

The relative quantitative real-time PCR (qPCR) was performed in a “Real Time PCR” thermocycler (Applied Biosystems, model 7500, Life Technologies), using a non-specific detection sequence (fluorophore) SYBR Green I. The abundance of transcripts of enzymes of fermentative pathways has been examined using specific primers, as shown in Table 4, drawn from the analysis of gene sequences already known in the library of *T. cacao*. In order to assess the quality of these primers, as well as the specificity and identity of reverse transcription products, the qPCR products were monitored after each PCR cycle using an analysis curve of reaction products that could distinguish specific and non-specific PCR products. The temperature of PCR products was elevated from 55 to 99°C, at a rate of 1°C/5 s, and the resulting data was assessed using LightCycler software. Only a single band with a characteristic melting temperature was observed for each sample, indicating that qPCR has produced a product that was specific for the used primers. In order verify whether the qPCR has only produced genes of interest, the PCR products were separated and visualized in 1% agarose gel.

**Table 4:** Pairs of gene-specific primers used in qPCR analysis.

Gene	Primer
ADH	Forward: 5′- GACGCCATATGTGTGAGCTGA-3′
	Reverse: 5′- ACGCGGGTTCTCTTATTAAAGGT-3′
PDC	Forward: 5′- TTGAGATTCATGATGGCCCTT-3′
	Reverse: 5′- CTCCTGTTGCTGTGGATATTG-3′
LDH	Forward: 5′-TGGCTACTCCGTGGCTAGCT-3′
	Reverse: 5′- AAGACGCCACCCCTACCAAG-3′
Beta-tubulin	Forward: 5′-TGCAACCATGAGTGGTGTTCA-3′
	Reverse: 5′-CAGACGAGGGAAGGGAATGA-3′

For the synthesis of double-stranded cDNA, the following method has been applied: 1 cycle at 50°C, for 2 min., 1 cycle at 95°C, for 10 min., 40 cycles at 95°C, for 15 s, and 60°C, for 60 s. The reaction mix consisted of 10 ng/µL of single stranded cDNA asa model, 0.5 µM of each primer, and a significant quantity of fluorophore SYBR Green I (Fermentas) in a final reaction volume of 25 µL.

Threshold cycle values (C_T_) were determined using the LightCycler software. For the purpose of detecting changes in the abundance of transcripts, numbers of the relative gene expression were calculated as a percentage of control plants using the 2 ^−**ΔΔCt**^ method [58] with the β-tubulin gene (Table 4) as endogenous (reference).

### Activity of fermentative enzymes

In order to analyze the activity of fermentation enzymes, such as ADH, PDC and LDH, samples of leaves and roots were collected 0, 3, 6, 12, 24, 48 and 96 h after the application of treatments. Enzymatic extract and activity readings were performed using an approach similar to that described by [36]. Initially, 3.0 g of plant tissue (roots and leaves) were macerated with liquid nitrogen and homogenized in 1 mL of a solution containing 100 mM Tris-HCl (pH 8.0), 10 mM Na-ascorbate, 10 mM DTT, 50 mg BSA and 15% (w:v) glycerol [59]. The solution was centrifuged at 30,000×*g*, for 20 min, and the supernatant was used in enzymatic assays.

The measurement of ADH activity was in the ethanol to acetaldehyde direction and was performed by monitoring the oxidation of NADH at 340 nm in a 350 µL reaction [60]: 85 mM MES (pH 6.5), 0.15 mM NADH, 50 µL of sample, and 5 mM acetaldehyde. The PDC activity was determined in a coupled reaction system in 350 µL of reaction buffer, as described by [61]: 85 mM MES (pH 6.5), 0.5 mM thiamine pyrophosphate, 0.5 mM MgCl_2_, 0.15 mM NADH, 14 units of ADH, 50 µL of sample and 10 mM sodium pyruvate. The measurement of LDH activity was in the pyruvate to lactate direction and was performed by monitoring the oxidation of NADH at 340 nm in the presence of 4-*methylpyrazole* to inhibit the interference of the coupled PDC and ADH reaction [59]. The reaction for the LDH assay was 350 µL containing 100 mM TES (pH 7.0), 0.15 mM NADH, 1 mM 4-methylpyrazole, 3 mM NaCN, 10 mM sodium pyruvate and 50 µL of sample. The activity of the three evaluated enzymes was expressed in absorbance units (AU) mg^−1^ dry matter (DM) h^−1^.

### Extraction of proteins

**Figure 7:**
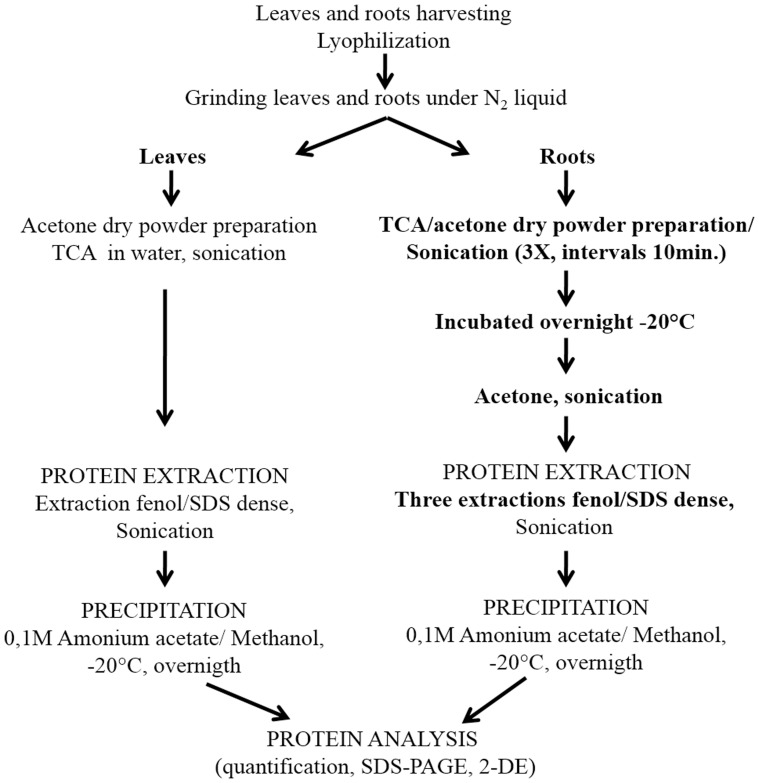
Scheme showing the protein extraction of the leaves and roots.

The total protein extracts from leaves and roots of flooded and control plants were collected 96 h after the application of treatments and were obtained by phenol extraction followed by precipitation with 0.1M ammonium acetate in methanol, as described by [62] for leaves, and [63] for roots (Fig. 7). Initially, 1.0 g of plant tissue (lyophilized roots) was pounded into a fine powder in liquid nitrogen using a pestle and mortar, and intermixed with 7% w/w polyvinylpolypyrrolidone (PVPP). The fine powder was re-suspended in 10 mL of 10% TCA in acetone (cold) and 0.07% 2-mercaptoethanol and was sonicated on ice (3 pulses of 5 s, 70% output, with 10 s intervals) on the Ultrasonic processor (Gex 130, 130 W). This process was repeated four times at intervals of 10 min. The mixture was incubated overnight, at −20°C, for complete precipitation of proteins. The mixture was then centrifuged at 10000×*g*, 4°C, for 10 min. The supernatant was discarded and the pellet was washed tree times with the same volume of cold acetone and 0.07% 2-mercaptoethanol. After that, the pellet was completely re-suspended by sonication on ice (3 pulses of 5 s, 70% output, with 10 s intervals), and then centrifuged at 10000×*g*, for 10 min, at 4°C. The final pellet was dried at room temperature, and whether used for protein extraction or stored at −20°C for future use.

The obtained pellet was re-suspended in 10 mL of dense SDS extracting buffer (30% sucrose, 2% SDS, 0.1 M Tris-HCl pH 8.0, 5% 2-mercaptoethanol). Then, the sample was submitted to sonication on ice (4 pulses of 10 s, 70% output, with 10 s intervals), and 5 mL of saturated phenol pH 8.0 was added. After that, the sample was centrifuged at 10000×*g*, 4°C, for 15 min. The supernatant was then transferred to a new tube and stored on ice. Proteins from the lower phase were twice extracted with a volume of dense SDS buffer and phenol. Phenol phases from the three extraction cycles were grouped and incubated overnight at −20°C, with five volumes of 0.1 M ammonium acetate in methanol. The pellet was generated through centrifugation at 10000×*g*, 4°C, for 20 min. Proteins were twice washed with 0.1M ammonium acetate in methanol, twice washed with acetone, and once washed with 80% ethanol; finally, the pellets were dried at room temperature. During every wash cycle, the sample was centrifuged at 10000×g, 4°C, for 5 min. The 2-D Quant kit (GE Healthcare) was used to determine protein concentrations, with bovine serum albumin used as standard. Three samples per treatment were prepared from three individual biological experiments. All samples were maintained at –80°C prior their use in electrophoresis.

### 2-D electrophoresis

The first-dimensional electrophoresis was carried out precast 13-cm IGP strips (pH 4–7, 130×3×0.5 mm; GE Healthcare, Little Chalfont, United Kingdom) using an Ettan IPGphor system (GE Healthcare). Total protein (350 µg) was mixed with rehydratation buffer [8 M urea, 2% (w/v) CHAPS, 18 mM DTT, 0.5% (w/v) IPG buffer (pH 4–7)] totaling 250 µL. Reydratation occurred at room temperature for 12 h on a ceramic plate (Manifold/GE). Afterwards, focusing was performed on the same apparatus, under the following conditions: step and hold at 500 V for 1 h, gradient 1000 V for 1 h, gradient 8000 V for 2∶30 h, and step and hold 8000 V for 55 min. After IEF, the strips were stored at −80°C until second-dimensional analysis. Before SDS-PAGE electrophoresis, the strips were incubated for 15 min in an equilibration buffer (6 M urea, 7.5 mM Tris-HCl pH 8.8, 29.3% glycerol, 2% SDS, 0.002% bromophenol blue) with 1% w/v DTT and, for another 15-min period, in an equilibration buffer with 2.5% w/v iodoacetamide. The strips were transferred onto vertical 12.5% SDS-PAGE gel. The second dimension (SDS-PAGE) was performed on a Ruby SE600 system (GE Healthcare): 15 mA, for 45 min, 40 mA, for 30 min, and 50 mA *per* gel, for 3 h, for every strip, at a constant temperature of 11°C. A High-Range Rainbow Molecular Weight Marker has been used (GE Healthcare). All 2-D gel separations were repeated three times. After electrophoresis, proteins were visualized with 0.08% w/v colloidal Coomassie G-250 [64]. Gels were scanned via ImageScanner II (Amersham) and analyzed using the ImageMaster 2D Platinum 6.0 software (GE Healthcare). Control group gels were compared with the flooded group gels (three independent biological replicates, three technical replicates). Differentially acquired protein spots were compared to find common differential protein spots. The spots were quantified based on the relative volume; t-test was performed and data were expressed as mean ± SD. P values less than 0.05 were regarded as statistically significant. Protein spots achieving a ≥ twofold increase en spot intensity and observed in three replicated gels from independent experiments were scored and subject to mass spectrometry.

### Mass spectrometry analysis

The selected protein spots were excised from the 2-DE gel, and destained with 50% acetonitrile containing 25 mM ammonium bicarbonate. The gel plugs were dehydrated with 100% acetonitrile, vacuum dried, digested with 4 µL of 25 ng/µL Promega Trypsin Gold (Mass Spectrometry Grade) on 25 mM ammonium bicarbonate, and incubated overnight at 37°C. The supernatant was collected and transferred to a new tube. The gel fragments were washed with 50% acetonitrile and 5% formic acid, and the supernatant was collected [65]. The supernatant obtained in the last two steps were pooled and concentrated under vacuum to a volume of 15 µL [66].

The resulting peptides from the digests were subjected to online nanoflow liquid chromatography tandem mass spectrometry [LC–MS/MS) on a nanoAcquity system (Waters, Milford, MA) coupled to an Q-ToF micro mass spectrometer (Waters). Peptide mixtures were loaded onto a 1.7-µm×100-mm nanoAcquity UPLC BEH300 column packed with C18 resin (Waters) and were separated at a flow rate of 0.6 µl.min^−1^ using a linear gradient of 1 to 50% solvent B (95% acetonitrile and 0.1% formic acid) in 23 min, followed by an increase to 85% solvent B in 4 min and held at 85% solvent B for additional 3 min. Solvent A was 0.1% formic acid inwater. The eluant from the high pressure liquid chromatography (HPLC) column was directly electrosprayed into the mass spectrometer, which operated in a data-dependent acquisition mode to automatically switch between full scan MS and MS/MS acquisition [65].

The MS data analysis was performed using the Masslynx 4.0 software (Waters- Micromass, Manchester, UK). Protein identification was performed using a Mascot server (Version 2.1.04, Matrix Science) and the *T. cacao* genome database [68]. The search criteria used were trypsin digestion, variable modifications set as cysteine (Cys) and methionine (Met), max of one missed cleavages allowed and peptide mass tolerance of ±0.3 Da for the parent ion and 0.10 Da for the fragment ions [66], [67].

### Statistical analyses

The experimental design was completely randomized, with four treatments relative to the two clonal genotypes of cacao and the two hydric regimes (control and flooded), four replicates and one plant per experimental unit, during seven collection periods (0, 3, 6, 12, 24, 48 and 96 h after application of treatments). The experimental results were subjected to analysis of variance (ANOVA). Tukey’s multiple comparison tests were performed to assess the differences between means (P<0.05). In order to test changes in the pattern of protein expression between control and flooded treatments, Student’s t-test has been employed (P<0.05) with three replications.
